# Supplementary figures and images for: Unlocking the potential of ancient hexaploid Indian dwarf wheat, Tritium sphaerococcum for grain quality improvement
Source: PeerJ. 2023 Jul 27;11:e15334. doi: 10.7717/peerj.15334 (PMC10387235; doi:10.7717/peerj.15334)

**
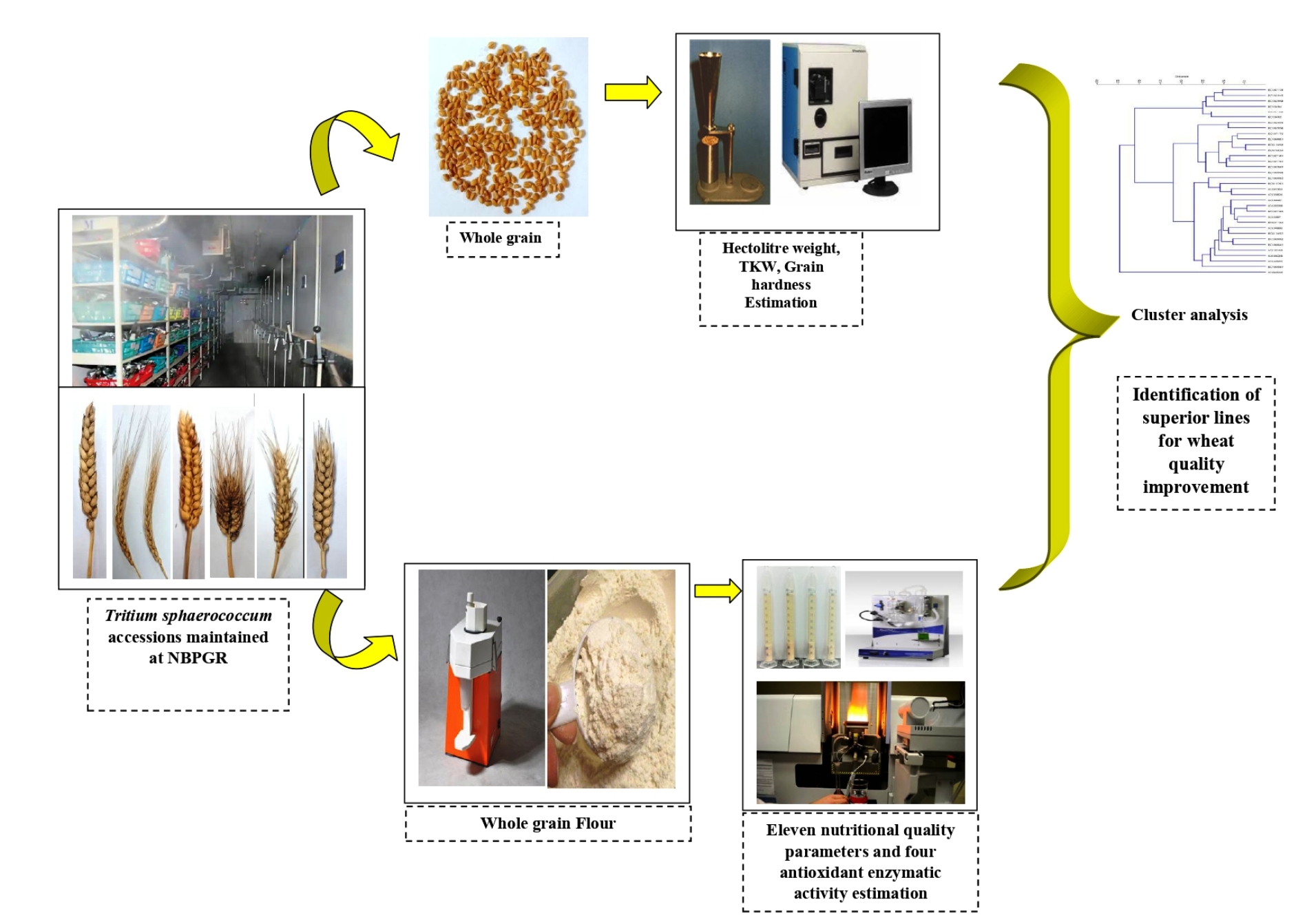
**

**Supplementary Fig. 1. Flow of work done for grain quality analysis**

Supplement: Supplemental Information 2 [file peerj-11-15334-s002.docx]
