# Supplementary material for: Unlocking the potential of ancient hexaploid Indian dwarf wheat, Tritium sphaerococcum for grain quality improvement
Source: PeerJ. 2023 Jul 27;11:e15334. doi: 10.7717/peerj.15334 (PMC10387235; doi:10.7717/peerj.15334)
Supplement: Supplemental Information 3 — a) Hectolitre weight (HW), b) Thousand grain weight (TGW) c) Sedimentation value (Sed), d) Hardness index (HI), e) Protein content (Pro), f) Albumin content (Alb), g) Globulin content [file peerj-11-15334-s003.docx]

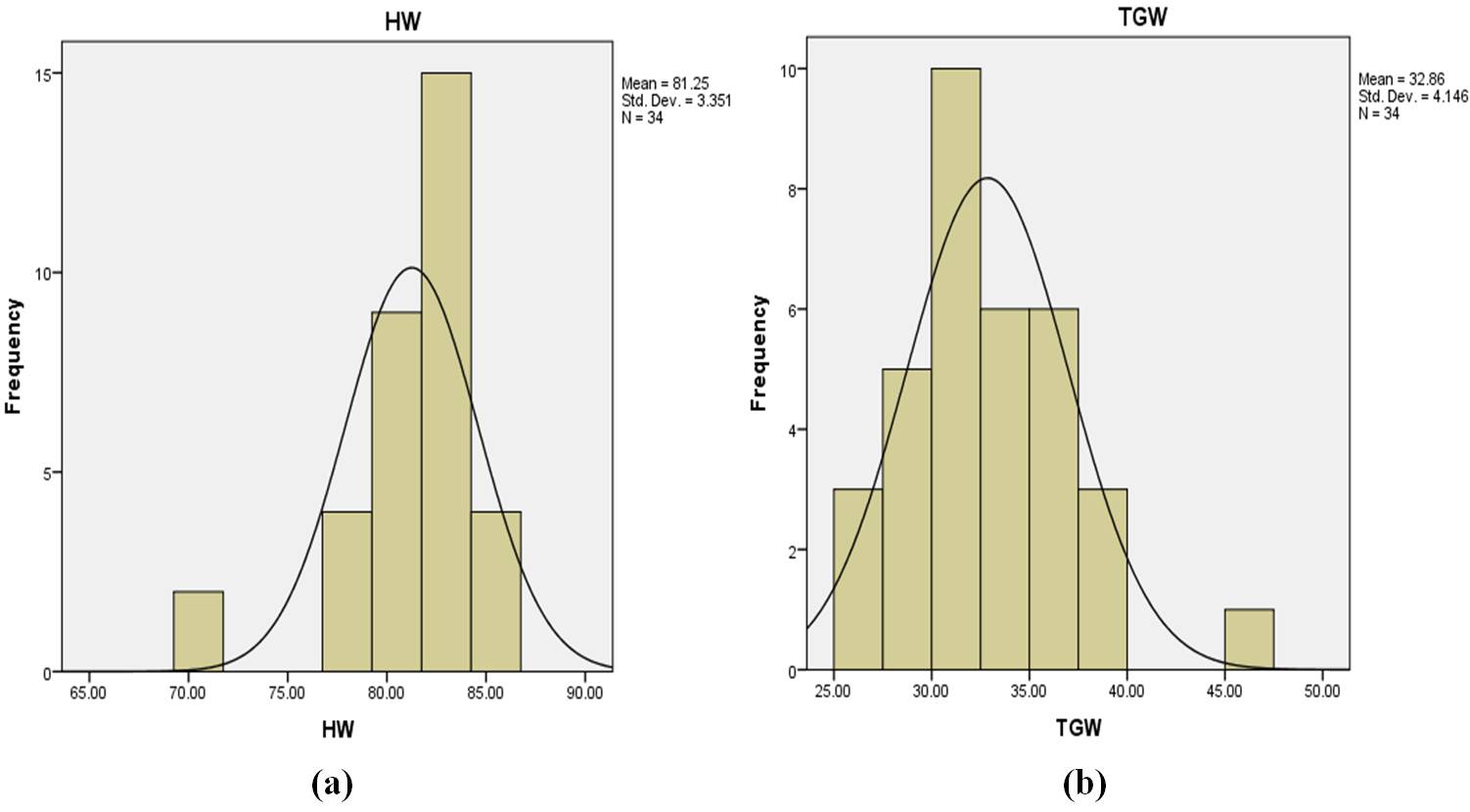


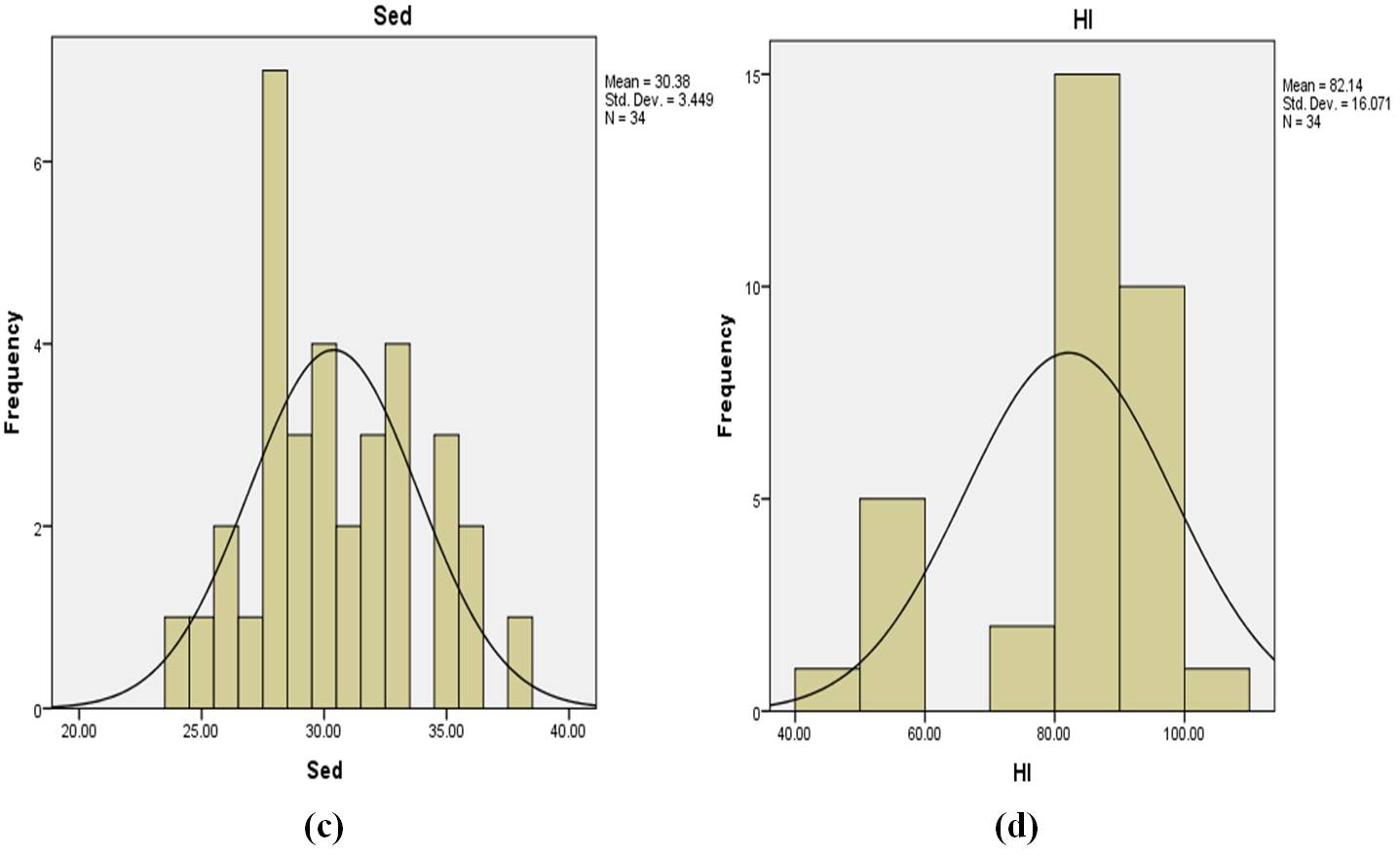


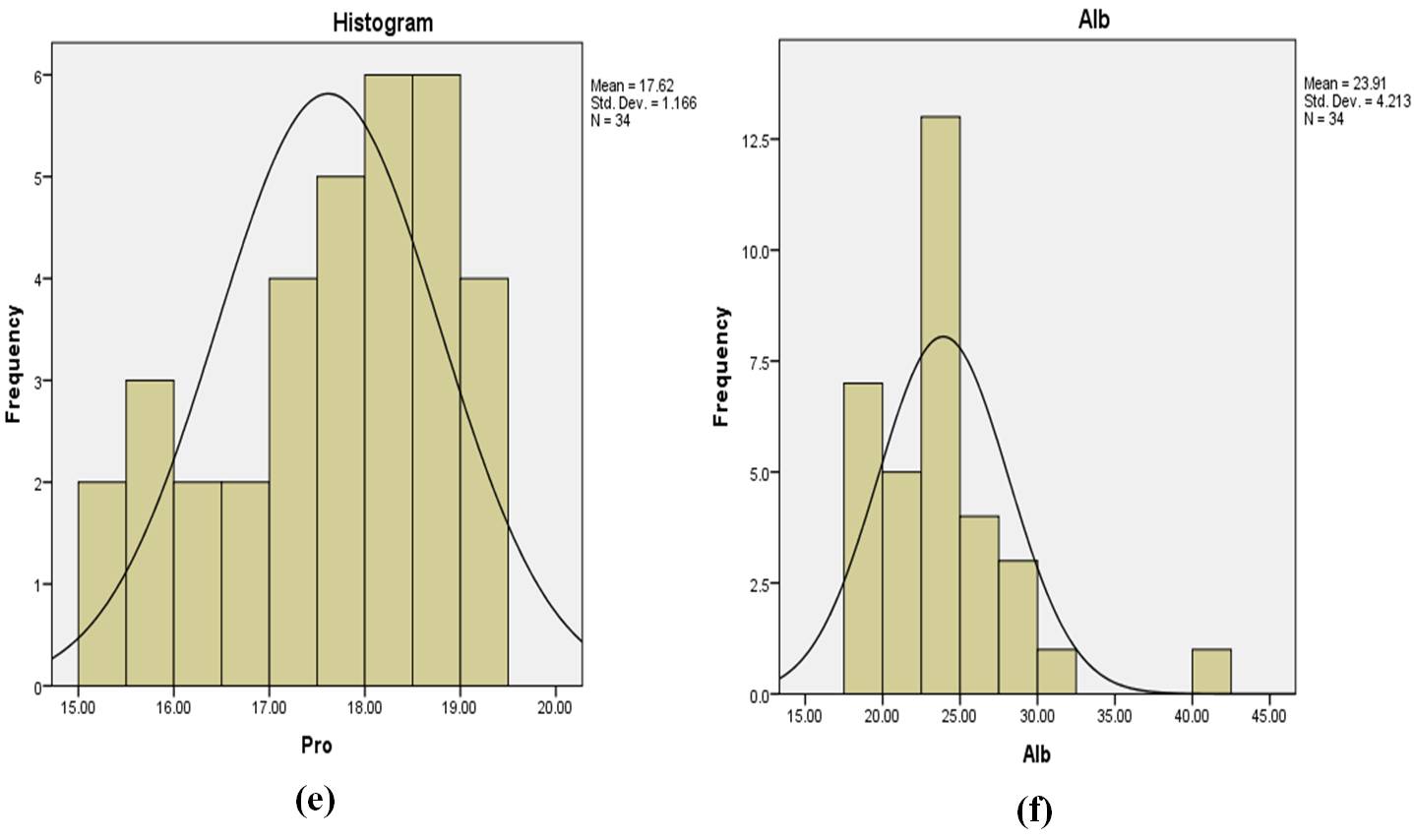


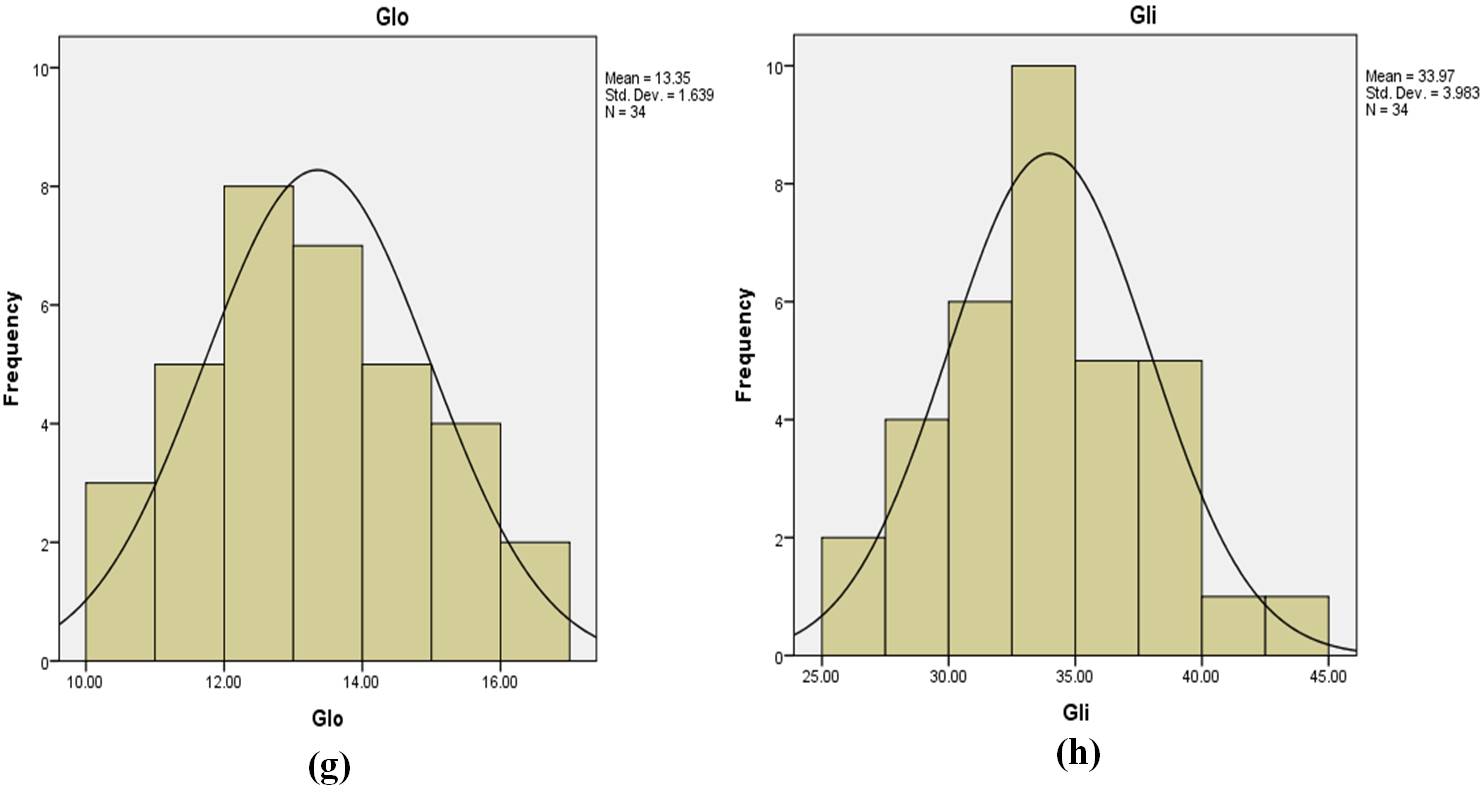


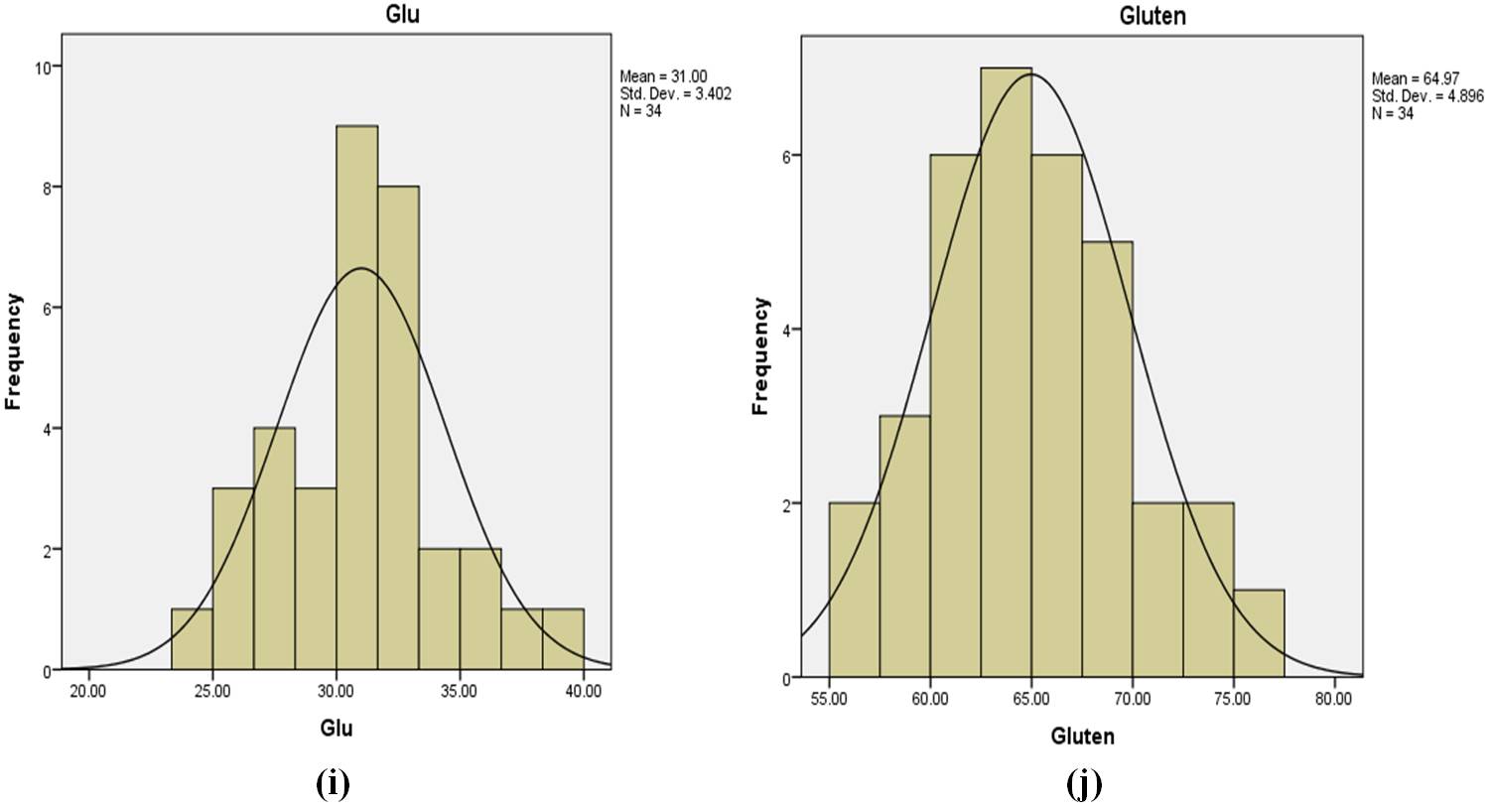


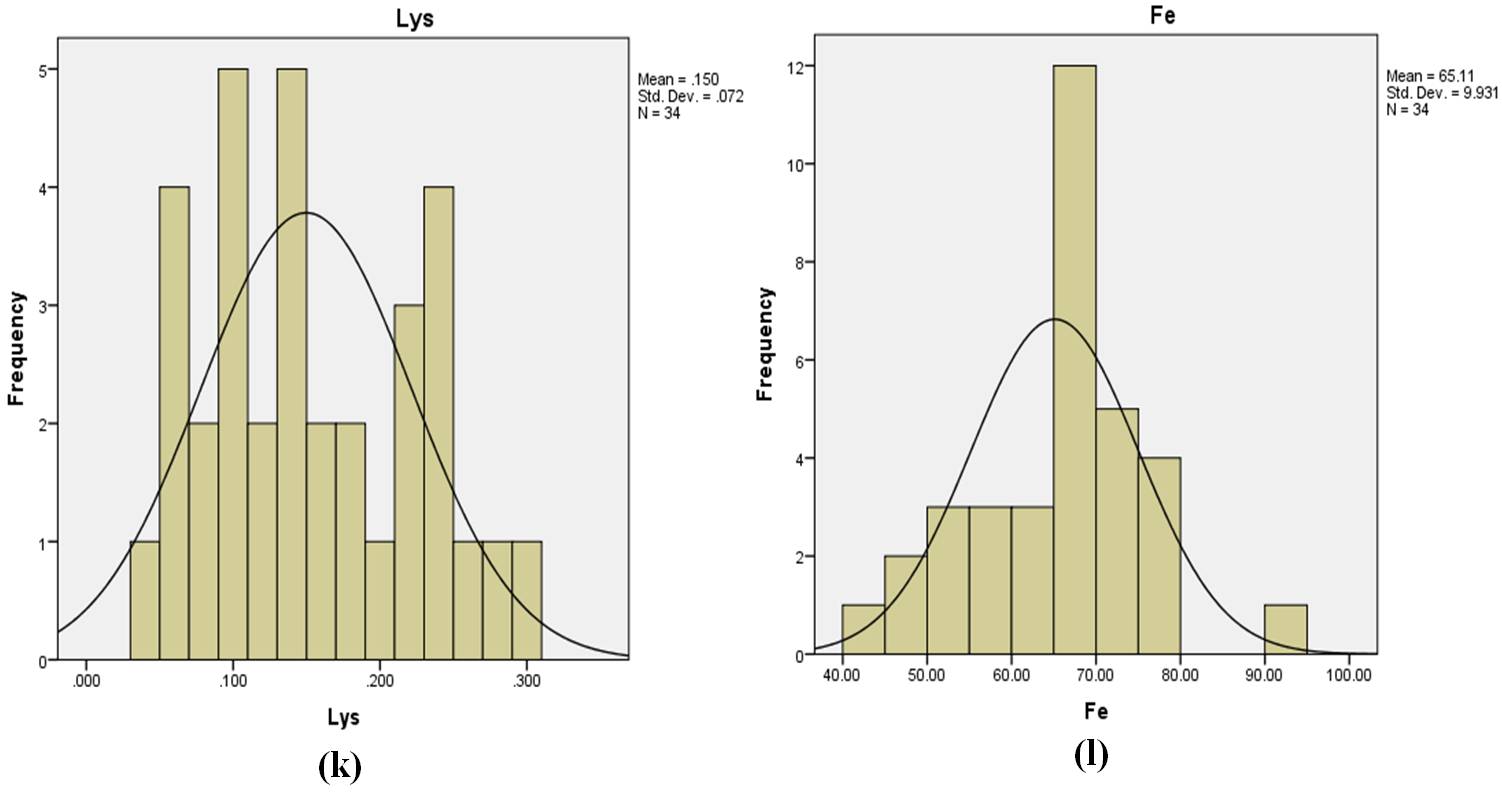


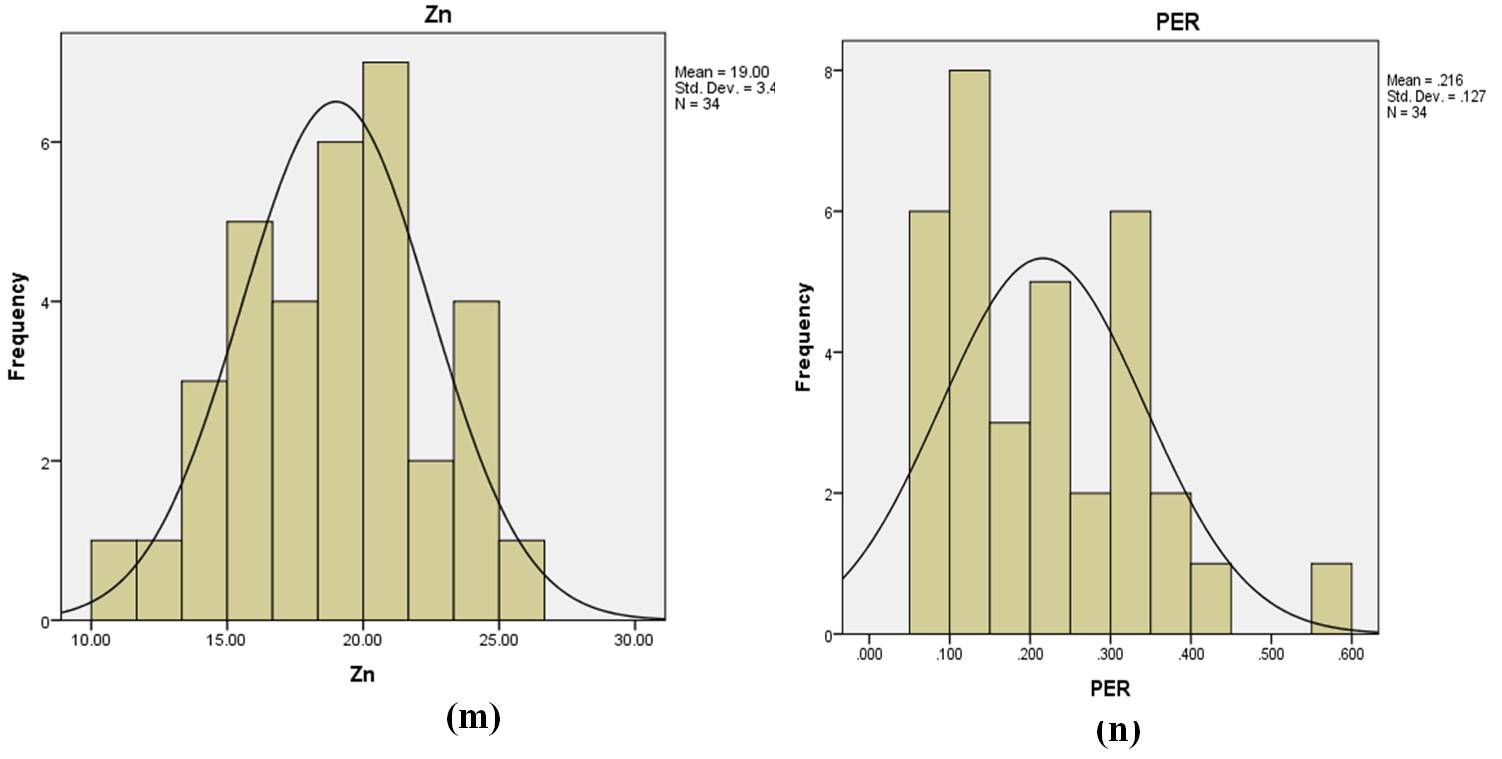


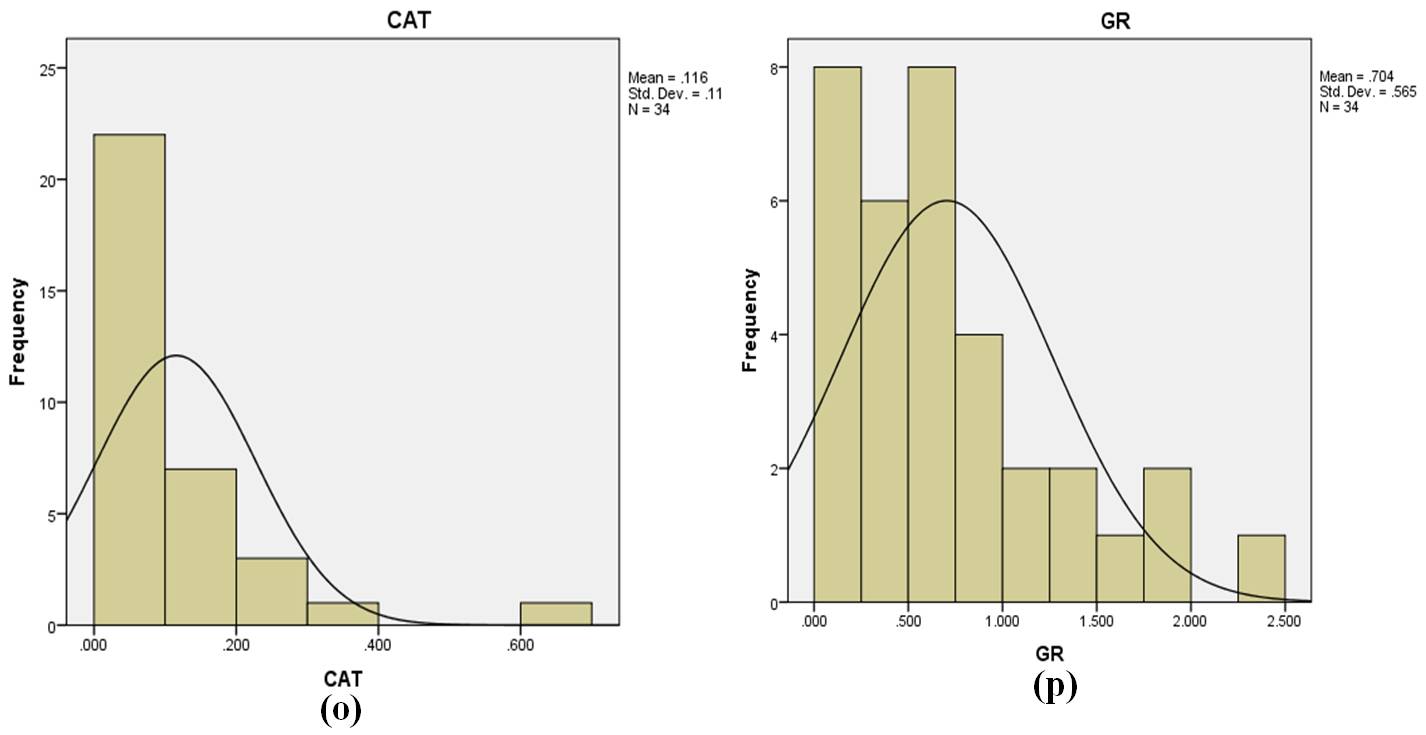

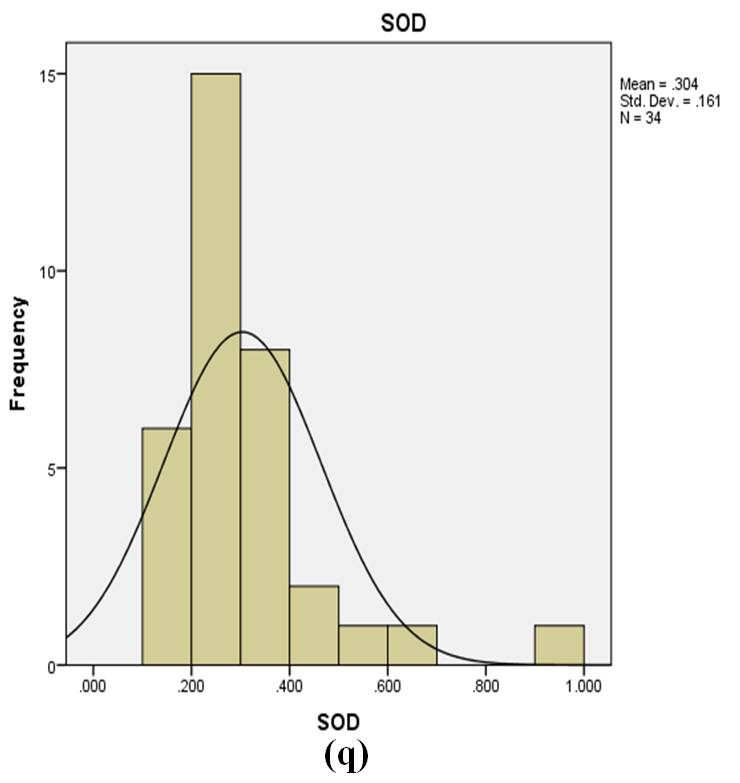


**Supplementary Fig. 2 Histogram depicting variations in different grain quality traits studied a) Hectolitre weight (HW), b)Thousand grain weight (TGW) c) Sedimentation value (Sed), d) Hardness index (HI), e) Protein content (Pro), f)Albumin content (Alb), g) Globulin content (Glo), h) Gliadin content (Gli), i) Glutenin content (Glu), j) Gluten content, k) Lysine content (Lys), l) Fe^+2^ Content, m) Zn^+2^ content, n) Peroxidase (POX), o) Catalase (CAT), p) Glutathione reductase (GR), q) Superoxide dismutase (SOD)**
